# Swimming expertise is associated with enhanced suprasecond visual duration discrimination across silent and rhythmic contexts

**DOI:** 10.3389/fpsyg.2026.1811223

**Published:** 2026-04-29

**Authors:** Yi Liu, Yunhao Zhang, Ye Mao, Senlin Lan, Yue Zhang, Haoping Yang, Yi Peng

**Affiliations:** 1 School of Education, Beijing Sport University, Beijing, China; 2 School of Psychology, Beijing Sport University, Beijing, China; 3 School of Competitive Sports, Beijing Sport University, Beijing, China

**Keywords:** competitive swimming, decision policy, duration discrimination, rhythmic sound, seconds range timing, sport expertise

## Abstract

**Background and aims:**

Whether athlete advantages in temporal processing primarily reflect sport-specific temporal knowledge or transfer to neutral laboratory judgments remains unresolved. Competitive swimming offers a theoretically informative case because successful performance depends on the precise regulation of pace and rhythm across repeated actions in a sensory-constrained environment. The present study examined whether swimming expertise is associated with superior performance in a neutral visual suprasecond duration-discrimination task and whether any expertise-related advantage remains observable across sensory contexts.

**Methods:**

Two independent experiments were conducted, each including 20 Expert Swimmers and 20 Amateur Controls. Participants completed a visual two-alternative forced-choice duration-discrimination task in which task difficulty was manipulated by the between-interval duration difference (200, 500, 1,000, and 2,000 ms). Experiment 1 was administered under silent visual conditions, whereas Experiment 2 used the same visual task while presenting task-irrelevant rhythmic auditory stimulation concurrently with each visual interval. Accuracy and reaction time (RT) were analyzed separately within each experiment.

**Results:**

In both experiments, discrimination accuracy increased as task difficulty decreased, confirming the effectiveness of the difficulty manipulation. Across difficulty levels, Expert Swimmers showed higher accuracy than Amateur Controls under both silent and task-irrelevant rhythmic conditions. Evidence for a larger expert advantage under higher difficulty emerged in Experiment 1 but was not reproduced in Experiment 2. Reaction-time findings did not indicate a generalized speed advantage, although difficulty-related variation in response patterning was observed. The rhythmic auditory background did not materially alter the overall accuracy advantage shown by Expert Swimmers.

**Conclusion:**

Swimming expertise was associated with enhanced performance in a neutral visual suprasecond duration-discrimination task across two sensory contexts. These findings indicate that the observed advantage was expressed primarily in discrimination accuracy rather than generalized response speed and remained observable when the task was embedded in a task-irrelevant rhythmic context. More broadly, the results support a bounded-transfer interpretation: swimmer expertise appears to extend beyond overtly sport-specific temporal content, but the present evidence does not support claims of unrestricted domain-general timing.

## 1 Introduction

When people judge whether a traffic light has taken unusually long to change or compare whether one event lasted longer than another, they rely on interval timing. This capacity supports temporal judgment, action coordination, and temporal preparation in everyday behavior (Buhusi and Meck, 2005; Coull et al., 2011; Grondin, 2010). Its relevance becomes especially pronounced in sport, where successful performance depends on the continuous regulation of pace and rhythm across repeated actions. This is particularly true in closed-skill sports, which are performed in relatively stable environments and permit substantial self-paced control (Brady, 1995; Wang et al., 2013). However, recent sport psychology research suggests that the cognitive consequences of closed-skill practice depend not simply on the open- vs. closed-skill distinction, but also on the specific perceptual, attentional, and control demands of the discipline itself (Li et al., 2024). Competitive swimming is therefore a theoretically important case because performance depends on the precise temporal organization of repeated movement cycles, the regulation of segmental pace, and the maintenance of temporal consistency under accumulating fatigue.

Importantly, the timing demands of swimming are multi-scale rather than unitary. At a local level, swimmers must regulate the rhythm of successive stroke cycles. At a broader level, they must maintain target split times and distribute effort across laps and race segments. Recent sports-science evidence shows that swimming speed depends on the interaction between stroke rate and stroke length across race distances, while pace accuracy improves when swimmers receive timely feedback about their ongoing performance (Altavilla et al., 2018; Staunton et al., 2025). Accordingly, temporal control in swimming cannot be reduced either to a single subsecond motor interval or to whole-race chronometric time alone. Rather, it reflects the coordination of nested temporal processes, from ongoing rhythm regulation to short-segment pacing and longer-horizon race management. This multi-level temporal structure makes swimming especially suitable for investigating whether prolonged sport practice sharpens temporal processing beyond the immediate sport context.

Swimming is also theoretically distinctive among closed-skill sports because it is performed in an aquatic environment in which external sensory cues are attenuated, intermittently unavailable, or less behaviorally useful than in many land-based disciplines. As a consequence, swimmers may need to rely more heavily on internal rhythm, proprioceptive monitoring, and learned temporal regularities than athletes training in environments where external visual and auditory cues remain continuously accessible. This point is no longer merely intuitive. In a recent comparison of Expert Swimmers and runners, Perrone et al. (2023) reported that swimmers were more accurate and consistent in time-processing tasks and explicitly proposed that this advantage may be linked to the sensory-muffled characteristics of the aquatic environment. Moreover, because exercise and physical effort can themselves distort subjective time, swimming offers a particularly informative model for studying temporal processing under conditions in which athletes must preserve accurate pacing despite physiological load (Edwards et al., 2024; Goudini et al., 2024; Bartolini et al., 2025). For these reasons, swimming is not simply one more closed-skill discipline; rather, it provides a stronger and more specific test case for expertise-related transfer in temporal processing.

Against this background, the central theoretical question remains unresolved. Specifically, it is still unclear whether athlete advantages in timing primarily reflect domain-specific temporal knowledge acquired through repeated sport practice, or whether such expertise is associated with more general duration discrimination abilities that transfer to neutral laboratory tasks (Perrone et al., 2023; Tobin and Grondin, 2012). One influential interpretation is the task duration knowledge account, according to which repeated engagement in highly practiced routines builds stable long-term knowledge about how long familiar actions should last (Tobin and Grondin, 2012). This account explains why swimmer advantages are often observed when experimental tasks overlap with practiced temporal structures or trained expectations (Bove et al., 2017). Indeed, previous studies have shown that swimmers are especially accurate when estimating the duration of familiar swimming actions, and that athletes' timing performance can be enhanced when the task preserves sport-related action structure, motor imagery, or implied movement content (Bove et al., 2017; Tobin and Grondin, 2012; Zheng, 2024).

At the same time, the literature also contains evidence pointing in the opposite direction. Chen and Cesari (2015), using neutral laboratory stimuli, reported that elite athletes produced more accurate and less variable time estimates than non-athletes, especially in the subsecond range. Likewise, Perrone et al. (2023) found that swimmers and runners outperformed non-athletes on time-processing tasks that were not explicitly sport-specific, with swimmers showing the strongest profile. These findings indicate that athlete timing advantages are not always restricted to overtly sport-linked materials. However, they still do not resolve the field's central contradiction. Chen and Cesari (2015) pooled athletes from different sport domains and used a reproduction paradigm with neutral scrambled-pixel stimuli, which is informative about general temporal estimation but does not isolate visual comparative discrimination in a specific sport population. Perrone et al. (2023), in turn, employed a broader battery including reproduction, finger tapping, and motor imagery, thereby demonstrating temporal benefits beyond direct sport simulation but leaving open whether those benefits extend to a neutral visual duration-discrimination task that minimizes motor production and practiced action schemas. Conversely, many studies that report athlete advantages have used implied-motion or imagery-based paradigms, where performance may be facilitated by perceptual familiarity, embodied simulation, or sport-specific sensorimotor knowledge rather than by a broader comparative timing ability (Bove et al., 2017; Zheng, 2024). Therefore, the available evidence remains theoretically mixed: there is support for both transfer and task specificity, but the mechanisms underlying this pattern have not yet been cleanly separated.

To address this problem, the present study uses the term domain-general duration discrimination ability in a deliberately restricted sense. Here, “domain-general” does not mean a fully amodal, modality-independent internal clock that is equally expressed across all sensory channels and all stimulus classes. Such a strong claim would be difficult to sustain, given extensive evidence that duration discrimination shows marked modality-specific and stimulus-specific characteristics, including robust visual-auditory differences across temporal ranges (Rammsayer et al., 2015; Cantarella et al., 2024). Instead, in the present study, domain-generality is operationalized conservatively as expertise-related transfer from swimming to a neutral, non-sport, non-motion, non-imagery visual comparison task that requires comparative temporal judgment rather than overt motor production. Under this definition, superior performance by Expert Swimmers on the present task would indicate transfer beyond practiced action semantics and beyond direct embodied familiarity with sport-specific stimuli. A stronger, but still bounded, form of this claim is tested in Experiment 2: namely, whether any swimmer advantage remains stable when the same neutral visual comparison is embedded in a different sensory context. Accordingly, the present design does not treat performance on a single visual task as exhaustive evidence for a fully domain-general timing faculty; rather, it tests a circumscribed and theoretically transparent form of generalization that is stronger than task duration knowledge, but weaker than full cross-modal amodality.

The broader timing literature further indicates that this issue cannot be resolved without considering the mechanisms engaged by the task itself. Scalar Expectancy Theory predicts that timing variability scales with interval length (Gibbon, 1977; Gibbon et al., 1984), whereas attentional accounts propose that subjective duration depends on the allocation of cognitive resources during the interval (Zakay and Block, 1997). Consistent with this view, meta-analytic evidence shows that cognitive load systematically alters duration judgments, indicating that timing performance is shaped not only by elapsed time but also by attention and task demands (Block et al., 2010). This distinction is especially important because the present study operates in the seconds-range (suprasecond) domain. Timing in this range is more strongly influenced by attention, working memory, temporal learning, and embodied or sensorimotor processes than very brief interval processing, which is more tightly constrained by low-level sensory factors (Rammsayer et al., 2015; Teghil and Wittmann, 2025). The present 2–7 s interval range therefore does not attempt to reproduce the full duration of a swimming race, nor the finest micro-temporal properties of a single stroke phase. Instead, it targets an intermediate temporal window that is theoretically closer to ongoing rhythm regulation and short-segment pacing control than to either ultra-brief sensory timing or whole-race chronometric estimation. In this respect, the chosen range is not a literal copy of swimming competition timescales, but a theoretically motivated compromise: it retains relevance to swimming-related temporal control while minimizing overt sport-specific content.

These considerations motivate the present methodological choices. First, the study uses a two-alternative forced-choice duration-discrimination task in which participants compare two successive visual intervals and decide which lasted longer. Because this paradigm targets comparative temporal judgment directly while minimizing action meaning, implied motion, and overt motor imagery, it offers a more conservative test of transfer than paradigms built around sport-specific movement content (Grondin, 2010). Second, task difficulty is manipulated through Δ*t*, defined here as the absolute difference between the two durations within a trial; in other words, Δ*t* indexes how difficult it is to discriminate the two intervals, with smaller Δ*t* values reflecting more difficult comparisons. In the present study, Δ*t* ranges from 200 to 2,000 ms, thereby providing a graded manipulation of discriminability across trials. Importantly, although the difficulty manipulation is expressed in milliseconds, the absolute durations remain in the seconds range (Experiment 1: 2,000–7,000 ms; Experiment 2: 2,000–7,000 ms). Consequently, the paradigm is more accurately described as a seconds-range duration-discrimination task with millisecond-scale manipulation of discriminability, rather than as a millisecond timing task. This distinction matters because it aligns the task with the temporal scale most relevant to suprasecond cognitive timing while preserving fine-grained experimental control. In addition, because the task yields both accuracy and reaction time (RT), it permits an assessment of whether expertise is expressed only in discrimination success or also in the speed-accuracy trade-off under graded uncertainty (Heitz, 2014).

The use of neutral visual stimuli also requires explicit justification. A central limitation of much of the existing athlete timing literature is that movement-related or expertise-related stimuli can confound temporal sensitivity with semantic familiarity, action prediction, or embodied simulation. This concern is especially relevant because recent evidence indicates that visual movement can impair duration discrimination at short intervals rather than facilitate it, meaning that dynamic displays may introduce perceptual variance unrelated to the theoretical question of interest (Torres et al., 2024). By using neutral visual intervals, the present study reduces these confounds and tests whether swimmer expertise transfers to comparative timing when the stimuli themselves carry no sport-related meaning. This allows any group difference to be interpreted more cautiously as evidence of transfer within visual comparative timing, rather than as a by-product of motion-specific processing.

Sensory context constitutes a further theoretical issue and directly motivates Experiment 2. Psychophysical research frequently reports higher temporal sensitivity in audition than in vision, including in the seconds range (Rammsayer et al., 2015; Cantarella et al., 2024). However, this does not justify the stronger assumption that rhythmic sound should automatically improve visual timing. On the one hand, rhythmic stimulation can entrain temporal attention when it is predictive or task-relevant, thereby shaping when attention is allocated in time (Lakatos et al., 2008; Nobre and van Ede, 2018). On the other hand, these effects depend on task structure and attentional set. Rhythmic sound does not reliably facilitate concurrent visual performance when attention is not directed to the auditory stream (De Winne et al., 2022), and task-irrelevant sounds can capture attention and impair ongoing processing rather than support it (Marois et al., 2019; Parmentier, 2014). Recent evidence further suggests that sensory entrainment can improve audiovisual temporal acuity under specific conditions, but not indiscriminately across contexts (Marsicano et al., 2024). Therefore, auditory rhythm should be treated as a theoretically meaningful boundary condition, not as a guaranteed performance enhancer. In the present study, Experiment 2 introduces rhythmic auditory stimulation precisely to test whether any swimmer advantage observed in a neutral visual comparison task remains stable, changes, or disappears when the same basic temporal judgment is placed in a different sensory context.

Building on this rationale, the present work reports two independent experiments using the same neutral visual comparison paradigm under two sensory contexts. Experiment 1 uses a purely visual sequential comparison. Experiment 2 preserves the same visual structure but embeds each visual interval in rhythmic auditory stimulation. The two experiments are treated as independent tests rather than as a direct cross-experiment comparison because they involve different participant samples. This design addresses a specific gap in the literature: prior work has not clearly determined whether Expert Swimmers outperform Amateur Controls on the same neutral seconds-range visual duration-discrimination task, whether such an advantage is observable when action semantics and implied motion are minimized, and whether it remains robust across sensory context while measuring both accuracy and reaction time. Therefore, if Expert Swimmers outperform Amateur Controls in both experiments, the resulting pattern would be more consistent with expertise-related transfer beyond sport-specific temporal knowledge. By contrast, if the pattern changes across sensory contexts, the findings will clarify the theoretical boundary conditions under which athlete timing advantages generalize beyond practiced movement routines. Either outcome will sharpen the distinction between domain-specific temporal knowledge and more general comparative timing transfer.

### 1.1 Research questions and hypotheses

The present study examined whether swimming expertise is associated with superior performance in a neutral visual suprasecond duration-discrimination task, and whether any expertise-related advantage is observable across two sensory contexts. Because the two experiments involved independent samples, all predictions were evaluated separately within each experiment rather than through a direct cross-experiment comparison. Group (Expert Swimmers vs. Amateur Controls) was treated as a between-participants factor. Task difficulty was treated as a within-participants factor and was operationalized by the between-interval duration difference, with smaller duration differences indicating higher difficulty and greater temporal uncertainty.

H1: Within each experiment, Expert Swimmers will show higher duration-discrimination accuracy than Amateur Controls across difficulty levels.

H2: Within each experiment, accuracy will increase as task difficulty decreases in both groups.

H3: Within each experiment, the accuracy advantage of Expert Swimmers over Amateur Controls will be greater under higher difficulty conditions, where temporal discrimination is more uncertain.

H4: Within each experiment, reaction time will decrease as task difficulty decreases in both groups.

**RQ1 (robustness of the expertise effect across sensory contexts)**. When each experiment is considered separately, is the expertise-related accuracy advantage observable both in the silent visual context and in the task-irrelevant rhythmic context?

**RQ2 (group differences in reaction-time patterning across difficulty)**. Does the relation between Group and reaction time vary across difficulty levels, consistent with possible group differences in response strategy under graded temporal uncertainty?

No directional hypothesis was specified regarding any additional facilitative effect of rhythmic sound, because in the present design the auditory sequence was task-irrelevant and non-predictive rather than an explicitly attended temporal cue.

## 2 Experiment 1

### 2.1 Materials and methods

#### 2.1.1 Participants

We used G^*^Power 3.1.9.7 (Faul et al., 2007) to estimate the minimum sample size required to detect the target group-by-difficulty interaction in a mixed-design repeated-measures ANOVA with two groups and four within-subject levels. The analysis specified an effect size of *f* = 0.25 (Cohen, 1988), an alpha level of 0.05, statistical power of 0.95, a repeated measures correlation of 0.50, and a non-sphericity correction of 1. The result indicated a minimum total sample size of 36 participants. This target sample size is consistent with duration discrimination studies comparing athletes and non-athletes (Zheng, 2024).

To account for potential attrition during the practice or testing phases, Experiment 1 ultimately included 40 male participants, comprising 20 Expert Swimmers and 20 Amateur Controls. All participants were recruited from the undergraduate student body or university athlete population at Beijing Sport University. To minimize the potential influence of sex differences on duration judgment and to improve the internal consistency of between-group comparisons, the sample was restricted to males. A meta-analysis of duration judgment has shown that sex differences are generally small but statistically significant; therefore, the use of a single-sex sample helps reduce an additional source of variance (Block et al., 2000).

The mean age of the total sample was 19.09 ± 0.83 years. The expert swimmer group consisted of 20 male athletes with a mean age of 19.05 ± 0.79 years. Their years of systematic swim training averaged 10.41 ± 1.14 years, their weekly sport-specific training volume averaged 11.96 ± 1.41 h, and their primary-event World Aquatics Points (WA points) averaged 695.00 ± 29.52. The amateur control group included 20 male participants with a mean age of 19.14 ± 0.89 years. Their swimming experience averaged 1.73 ± 0.63 years, and their weekly general physical activity averaged 3.56 ± 1.55 h. Detailed sample characteristics for each group are presented in Table 1.

**Table 1:** Participant characteristics for Experiment 1.

Group	Age	Gender	Expertise		
			Experience (years)	Exercise (hours/week)	WA points
Experts	19.05 ± 0.79	20M	10.41 ± 1.14	11.96 ± 1.41	695.00 ± 29.52
Amateurs	19.14 ± 0.89	20M	1.73 ± 0.63	3.56 ± 1.55	–
Total	19.09 ± 0.83	40M	–	–	–

Values are reported as mean ± standard deviation (*M* ± *SD*). World aquatics points (WA points) index competitive swimming performance, with higher scores indicating higher performance; WA points are reported for expert swimmers only, all of whom met national-level criteria. Swimming experience (years) indicates years of involvement in swimming, and exercise (hours/week) indicates weekly physical activity (supervised swim training for expert swimmers vs. general activity for amateur controls, excluding any systematic swim training); Amateur Controls also reported no competitive swimming experience.

Participant screening followed a two-stage procedure. In the first stage, all candidates completed a structured screening questionnaire covering demographic information, handedness, visual and auditory status, training history, competition level, weekly training or physical activity volume, medical history, current medication use, and prior participation in similar experiments. In the second stage, conducted on the day of testing, the experimenter verified the questionnaire responses in person, confirmed the accuracy of group assignment, and recorded the demographic and background variables to be included in the data analysis, including age, handedness, visual and auditory status, years of systematic training, and weekly training volume. For the expert swimmer group, World Aquatics Points (WA points) and competition-level information were additionally verified. All participants provided written informed consent before the experiment began. The study was approved by the Ethics Committee of Beijing Sport University (Approval No. 2024349H) and was conducted in accordance with the ethical principles of the Declaration of Helsinki.

The inclusion criteria applied to both groups were as follows: male sex; age between 18 and 20 years; right-handedness; normal or corrected-to-normal vision; normal hearing; and the ability to understand and complete the experimental tasks independently. The exclusion criteria for both groups were: self-reported neurological disorders, major psychiatric disorders, or a history of concussion or residual symptoms following traumatic brain injury; current use of medication that could affect central nervous system function; uncorrected visual or auditory impairment; prior participation in highly similar time-perception experiments; and failure to meet the predetermined task qualification criterion during the practice phase.

Participants were included in the expert swimmer group if they were currently undergoing systematic swim training, were still actively competing in formal events, and had achieved at least the level of National First-Class Athlete, with eligibility for national-level competition in the current year. These qualifications were verified against official competition records. The amateur control group was permitted to have limited recreational swimming experience (≤ 2 years), but participants could not have any history of systematic swim training, any competitive swimming experience, or any specialized athletic training comparable in intensity to that of the expert group within the past year.

#### 2.1.2 Apparatus and stimuli

Experiment 1 employed a cross-sectional between-groups laboratory design to compare performance between Expert Swimmers and Amateur Controls on a purely visual suprasecond duration-discrimination task. To ensure that between-group differences would reflect the target cognitive processes as closely as possible, rather than situational noise, all participants completed the same task protocol under identical laboratory conditions, with equipment parameters, stimulus settings, and response mappings held constant across testing sessions. The experimental program was written in MATLAB 2021 and implemented using Psychtoolbox 3.1.7 (Kleiner et al., 2007). Visual stimuli were presented on a 24-inch LCD monitor with a resolution of 1,920 × 1,080 pixels and a refresh rate of 60 Hz. Screen luminance was calibrated to approximately 100 cd/m^2^. All testing took place in the same laboratory. During data collection, ambient illumination was maintained at 35 lux (measured at participants' eye level), background noise was kept at ≤ 35 dB(A) LAeq, and the laboratory was free of continuous conversation, telephone rings, or other salient transient sources of distraction. Participants were seated at a viewing distance of approximately 60 cm from the monitor. To minimize the potential influence of circadian rhythm and time-of-day variation on attentional state, response speed, and other basic cognitive processes, all sessions were conducted between 09:00 and 12:00 (Valdez, 2019; Munnilari et al., 2024). Responses were collected using the same standard keyboard in all sessions, with all participants pressing the F key with the left hand and the J key with the right hand. Stimulus presentation, temporal control, response recording, and trial-level data logging were fully automated, thereby reducing experimenter-dependent error and improving reproducibility.

The experimental background was black (RGB: 0, 0, 0). The visual stimuli consisted of two colored squares: a blue square (RGB: 0, 0, 255) and a yellow square (RGB: 255, 255, 0), each measuring 150 × 150 pixels. During the sequential presentation phase, a single square was always displayed at the center of the screen. During the choice phase, the two squares were presented simultaneously, with the blue square positioned on the left and the yellow square positioned on the right. The centers of the two squares were located 150 pixels to the left and 150 pixels to the right of the screen center, respectively. Experiment 1 did not include any auditory stimulation; all conditions were strictly visual-only. This configuration ensured that task demands were concentrated on visual duration information itself, thereby providing a relatively clean perceptual judgment framework for subsequent between-group comparisons.

#### 2.1.3 Procedure

Experiment 1 employed a single-session, two-alternative forced-choice visual duration-discrimination task (2AFC). Each participant took part in only one experimental session and completed the entire task individually. Because suprasecond duration judgments are particularly susceptible to strategic processes such as explicit counting, all participants received the same standardized verbal instructions before both the practice phase and the formal test. Specifically, they were instructed not to use overt counting, subvocal rehearsal, repeated words, internal rhythm, or any other artificial timing strategy during task performance, but instead to base their responses on their direct subjective impression of which interval lasted longer (Rattat and Droit-Volet, 2012; Riemer et al., 2022). This procedure was intended to minimize construct-irrelevant variance arising from strategic timing and to ensure that the formal assessment more closely reflected direct perceptual judgments of duration differences.

To ensure that all participants had fully understood the task requirements before formal testing, and to screen for clearly non-compliant response patterns in advance, the formal task was preceded by 10 practice trials. This arrangement was based on familiarization procedures used in previous duration discrimination studies of a similar type (Li et al., 2022). The practice trials were identical to the formal trials in terms of stimulus structure, presentation order, response mapping, and temporal sequence, but were not included in the final analysis. Trial-by-trial feedback was provided only during the practice phase to facilitate familiarization with the task rules and response procedure. In accordance with the laboratory's predefined quality-control criteria and prior principles for handling invalid responses (Skylark and Gheorghiu, 2017), participants did not proceed to the formal test if they produced fewer than five correct responses during practice or if their invalid response rate exceeded 10%, including anticipatory responses with reaction times shorter than 200 ms and omitted responses. The rationale for this procedure was that the practice phase served primarily to ensure task comprehension and adaptation to the procedure, and only secondarily as a data-quality screening step before formal testing.

The formal task used a fixed-trial design. Each trial followed the same temporal sequence. First, a central fixation cross (“+”) was presented for 500 ms. Next, the first square (either blue or yellow) was presented alone at the center of the screen for its preset duration. Immediately after it disappeared, the second square (the remaining color) was presented alone at the center of the screen for its preset duration. Importantly, the presentation order of the two colors was randomized on a trial-by-trial basis and strictly counterbalanced within each difficulty level. Once both sequential intervals had ended, the task entered the choice phase, in which the blue and yellow squares appeared simultaneously on the left and right sides of the screen, respectively, forming the response interface. Participants were required to indicate which square had lasted longer during the preceding single-square presentation phase. Pressing the F key indicated that the left blue square had lasted longer, whereas pressing the J key indicated that the right yellow square had lasted longer. The choice screen remained visible until a response was made or until the 4,000 ms response deadline was reached. The intertrial interval was 500 ms. No trial-by-trial feedback was provided during the formal test, so as to avoid introducing additional guidance that might influence subsequent judgment strategies.

Task difficulty was manipulated through Δ*t*, defined as the absolute difference in duration between the two temporal intervals. Four difficulty levels were used: Δ*t* = 200 ms, 500 ms, 1,000 ms, and 2,000 ms. Smaller Δ*t* values indicated finer interval differences and therefore greater discrimination difficulty, whereas larger Δ*t* values reflected more salient differences and thus lower task difficulty. Each difficulty level included 20 formal trials, yielding a total of 80 formal trials. Within each Δ*t* level, the presentation order (blue-first vs. yellow-first) and the target duration (blue-longer vs. yellow-longer) were fully counterbalanced. Trial order was randomized separately for each participant before the start of the experiment. On each trial, the two individual durations were generated within the allowable range of 2,000–7,000 ms, subject to the Δ*t* constraint for that trial, while ensuring that the longer interval did not exceed the upper limit. This design allowed task difficulty to be systematically manipulated within a constant perceptual judgment framework while reducing the likelihood that participants could adopt predictive strategies based on fixed duration pairings.

Data acquisition was fully automated. For each trial, the program recorded participants' basic information, including participant ID, sex, and age. It also logged the condition variables, including Δ*t*, the actual presentation duration of the blue square, the actual presentation duration of the yellow square, and which color had objectively lasted longer. In addition, the program recorded key temporal markers, including the onset times of the fixation cross, the blue square, the yellow square, and the choice screen. Each trial's keypress response, reaction time (RT), and accuracy were saved. RT was measured relative to the onset of the choice screen and recorded by the program in seconds, then converted to milliseconds before statistical analysis. If a participant pressed the Escape key during the choice phase, the task terminated immediately and all previously completed trial data were saved automatically. All raw data were exported as comma-separated values (.csv) files and simultaneously saved as backup MATLAB (.mat) files. Filenames included the experiment name, participant ID, and timestamp to ensure traceability of the raw data. By automating the entire recording process, the study further reduced the risk of bias arising from manual recording error and experimenter-related variability.

After completing the formal task, all participants filled out a brief written strategy check form to assess whether they had used counting, internal speech, subvocal singing, rhythmic repetition, imagined action repetition, or any other artificial timing strategy during the formal trials. The study specified *a priori* that any participant who explicitly reported sustained use of active counting during the formal task would be excluded from the analysis (Bangert et al., 2011; Allman et al., 2011). Together, the pretest no-counting instruction and the posttest strategy check formed a complementary strategy-control procedure: the former was intended to prevent strategic timing from occurring, whereas the latter served to identify and exclude any residual strategy contamination at the analysis stage. Because the task involved suprasecond intervals, strategic counting could not be fully ruled out. The no-counting instruction and post-task strategy check were used to reduce and monitor, rather than definitively eliminate, counting-related contamination.

**Figure 1:**
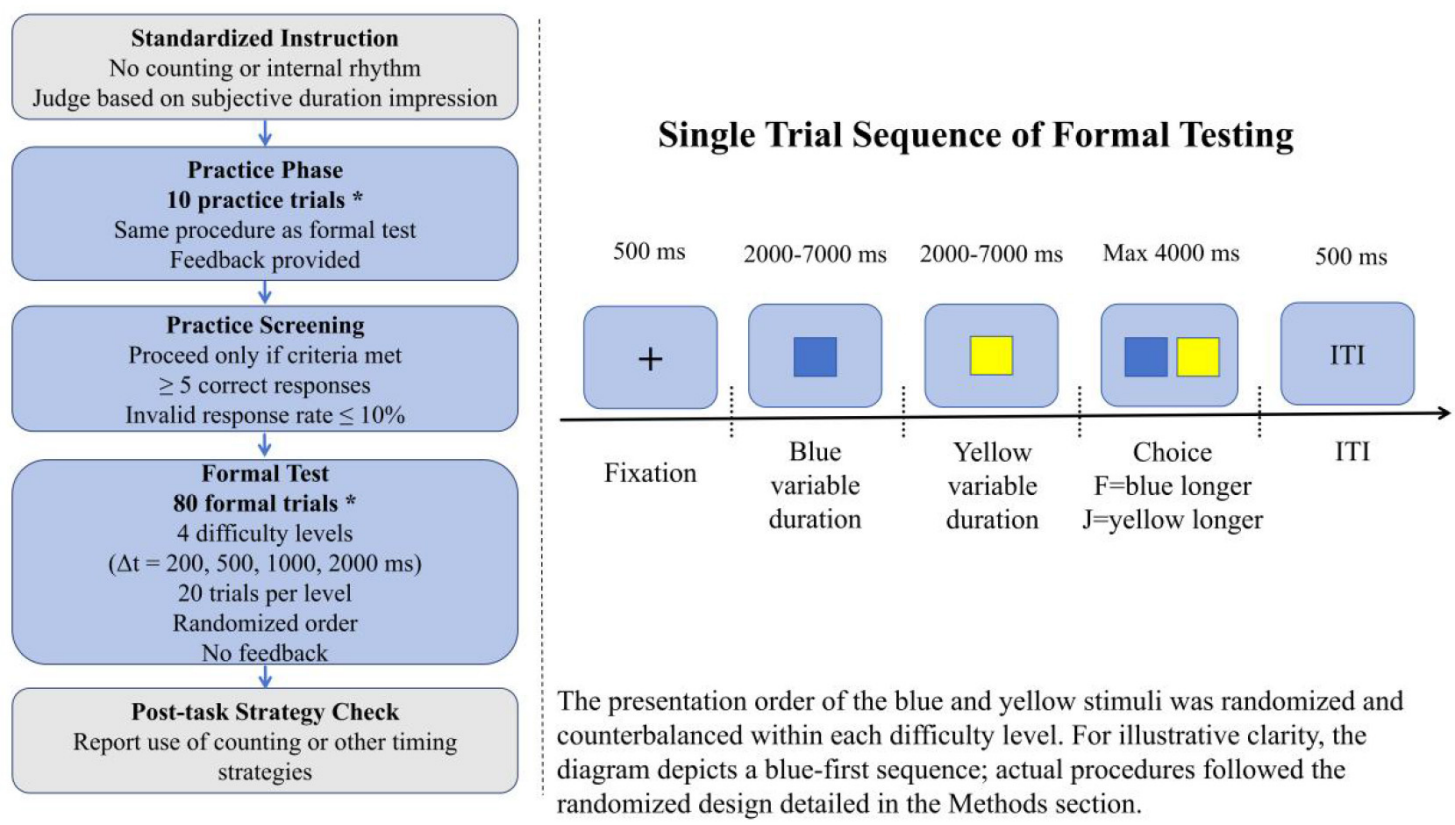
Procedure of Experiment 1 in the visual duration-discrimination task. After receiving standardized instructions, participants completed 10 practice trials with feedback and then proceeded to an 80-trial formal test comprising four difficulty levels (20 trials per level), with no feedback provided throughout formal testing. In each trial, participants viewed a 500-ms fixation cross, followed by sequential presentation of a blue square and a yellow square, each with a duration ranging from 2,000 to 7,000 ms, and then judged within 4,000 ms which square had lasted longer. The intertrial interval was 500 ms. A post-task strategy check was administered after completion of the formal test.

Several potential sources of bias were also controlled prospectively to improve scientific transparency. First, all participants were tested using the same equipment, the same response mapping, the same laboratory environment, and the same time window of day, thereby minimizing the influence of device differences, contextual variation, and time-of-day effects on behavioral performance (Valdez, 2019; Munnilari et al., 2024). Second, stimulus presentation, temporal control, response collection, and data storage were all automated, thereby reducing experimenter recording error and procedural variability. Third, the within-trial presentation order of the two stimuli was randomized. Importantly, the proportion of blue-first and yellow-first sequences was strictly counterbalanced within each difficulty level to eliminate potential interval-order effects (see Figure 1).

#### 2.1.4 Data analysis

All data preprocessing and statistical analyses were conducted in R 4.5.1. Data cleaning and reshaping were performed using the dplyr and tidyr packages. Mixed-design analyses of variance were conducted with the afex package, simple-effects analyses and *post hoc* comparisons were carried out using emmeans, and distributional diagnostics and effect-size estimation were performed using the moments, car, and effectsize packages, respectively. To maintain maximal consistency with the logic of SPSS output, analyses of variance were based on Type III sums of squares, and factors were coded using sum-to-zero contrasts. The afex package uses Type III sums of squares by default and is therefore well suited to an ANOVA framework aligned with that of commercial statistical software.

Prior to statistical analysis, raw behavioral data were preprocessed. At the participant level, individuals were excluded if their overall invalid response rate exceeded 10% or if their global accuracy fell below the 50% chance level. For the remaining sample, trial-level data cleaning was conducted. Specifically, trials with reaction times (RTs) shorter than 200 ms (anticipatory responses) or those reaching the 4,000-ms deadline (omissions) were classified as invalid and discarded. Finally, all subsequent RT analyses were restricted exclusively to valid trials with correct responses.

The distribution of each dependent variable was first assessed using the Shapiro–Wilk test. When the normality test indicated a significant deviation from normality, the extent of deviation was further evaluated in conjunction with skewness and kurtosis. Distributions were considered acceptably approximately normal when |skewness| <2 and |kurtosis| <7. These thresholds represent commonly used empirical criteria in applied research and help avoid overreliance on a single normality test, which can be overly sensitive when sample sizes are small.

Homogeneity of variance between groups was assessed using Levene's test, and the sphericity assumption for the within-subject factor was evaluated using Mauchly's test of sphericity. When the sphericity assumption was violated, Greenhouse–Geisser corrections were applied uniformly to adjust the degrees of freedom and corresponding significance tests. Because repeated-measures ANOVA is sensitive to violations of sphericity, reporting sphericity tests and any corresponding corrections was considered a necessary step for all within-subject main effects and interactions.

Accuracy and reaction time were analyzed separately using 2 × 4 mixed-design ANOVAs, with Group (Experts vs. Amateurs) as the between-subject factor and Task Difficulty (Δ*t* = 200 ms, 500 ms, 1,000 ms, and 2,000 ms) as the within-subject factor. The significance threshold was set at α = 0.05. For all main effects and interactions, *F*-values, degrees of freedom, *p*-values, η*p*^2^, and their 95% confidence intervals were reported. For planned comparisons and for the decomposition of significant interactions, simple-effects analyses were conducted. For between-group comparisons at a given difficulty level, independent-samples *t*-tests were used when the assumption of homogeneity of variance was met; when significant heterogeneity of variance was present, Welch's *t*-tests were used to obtain variance-robust estimates of the degrees of freedom and standard errors. Comparisons across difficulty levels within groups were conducted using paired-samples *t*-tests. All *p*-values for multiple comparisons were adjusted using the Bonferroni correction, and effect sizes for local comparisons were reported as Cohen's *d*.

### 2.2 Results

For Experiment 1, 50 individuals were initially screened. Of these, 2 were excluded as they failed to meet the study's prespecified inclusion criteria, 4 did not meet the practice phase qualification criteria (1 excluded for substandard overall accuracy in the practice phase, 3 for failure to meet the prespecified reaction time criteria), 1 was excluded because their overall accuracy in the formal task did not exceed chance level, 2 were excluded for failure to meet the prespecified reaction time criteria in the formal task, and 1 was excluded due to self-reported sustained active counting during the formal test. The final analyzed sample therefore comprised 20 expert swimmers and 20 amateur controls.

All analyses were based on 40 participants, including 20 Expert Swimmers and 20 Amateur Controls. Preliminary assumption checks indicated that the data were generally suitable for parametric analyses. Across all Group × Task Difficulty cells, skewness ranged from −1.891 to 0.189 and kurtosis ranged from −1.064 to 2.866 for accuracy, whereas skewness ranged from 0.071 to 1.701 and kurtosis ranged from −0.966 to 2.333 for reaction time. Mauchly's test indicated that the sphericity assumption was met for accuracy, *W* = 0.866, *p* = 0.384, but violated for reaction time, *W* = 0.714, *p* = 0.030; therefore, Greenhouse–Geisser correction was applied to the reaction time analyses. Levene's tests indicated homogeneous variances for accuracy at Δ*t* = 200 ms and 500 ms, but unequal variances at Δ*t* = 1,000 ms and 2,000 ms; accordingly, Welch-adjusted tests were used for the corresponding between-group follow-up comparisons. For reaction time, the homogeneity assumption was met at all four difficulty levels.

#### 2.2.1 Accuracy

Descriptive statistics showed that Expert Swimmers outperformed Amateur Controls across all four difficulty levels. In Expert Swimmers, mean accuracy was 0.628 (*SD* = 0.085) at Δ*t* = 200 ms, 0.822 (*SD* = 0.110) at Δt = 500 ms, 0.943 (*SD* = 0.059) at Δ*t* = 1,000 ms, and 0.984 (*SD* = 0.030) at Δ*t* = 2,000 ms. In Amateur Controls, the corresponding values were 0.548 (*SD* = 0.094), 0.626 (*SD* = 0.072), 0.743 (*SD* = 0.132), and 0.868 (*SD* = 0.097), respectively (see Table 2).

**Table 2:** Experiment 1 descriptive statistics for accuracy and reaction time.

Group	Δ*t* = 200 ms	Δ*t* = 500 ms	Δ*t* = 1,000 ms	Δ*t* = 2,000 ms
Accuracy (*n* = 20)				
Experts (*M* ±*SD*)	0.628 ± 0.085	0.822 ± 0.110	0.943 ± 0.059	0.984 ± 0.030
Amateurs (*M* ±*SD*)	0.548 ± 0.094	0.626 ± 0.072	0.743 ± 0.132	0.868 ± 0.097
Reaction time (*n* = 20)				
Experts (*M* ±*SD*)	1131 ± 388	1006 ± 304	866 ± 253	737 ± 224
Amateurs (*M* ±*SD*)	817 ± 306	841 ± 303	764 ± 258	684 ± 235

For the expert swimmer group and the amateur control group (*n* = 20 per group), data at each task difficulty level, indexed by Δ*t* (200, 500, 1,000, and 2,000 ms), are presented as mean ± standard deviation (*M* ± *SD*). All values are reported to three decimal places, and reaction times (RTs) are reported in milliseconds.

A 2 × 4 mixed-design repeated-measures ANOVA revealed a significant main effect of Group, *F*_(1, 38)_ = 94.023, *p* < 0.001, η*p*^2^ = 0.712, and a significant main effect of Task Difficulty, *F*_(2.753, 104.597)_ = 112.945, *p* < 0.001, η*p*^2^ = 0.748. The Group × Task Difficulty interaction was also significant, *F*_(2.753, 104.597)_ = 4.628, *p* = 0.006, η*p*^2^ = 0.109.

**Figure 2:**
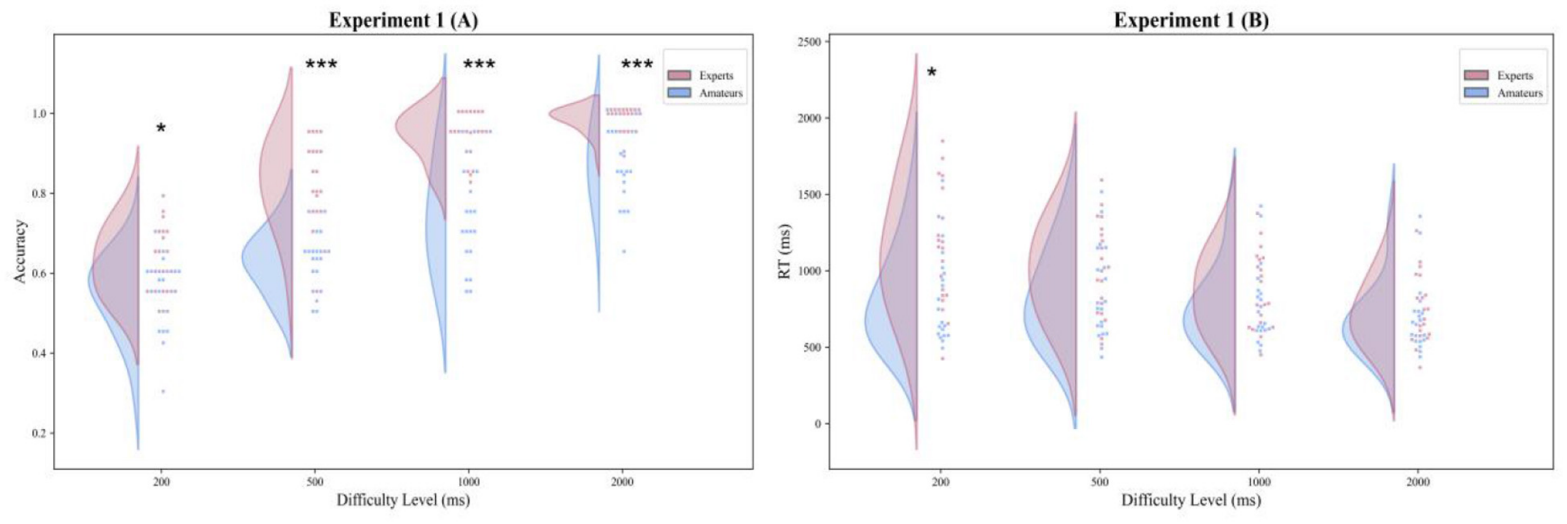
Experiment 1 (visual-only) performance for experts and amateurs (*n* = 20 per group) across Δ*t* (200, 500, 1,000, 2,000 ms). **(A)** Accuracy. **(B)** Reaction time (ms; from choice-display onset); asterisks indicate Bonferroni-corrected between-group differences at each Δ*t* (*p < 0.05, ***p < 0.001).

Because the interaction was significant, Bonferroni-adjusted simple-effects analyses were conducted. In the statistical file, the between-group contrasts were reported in the direction of Amateur Controls minus Expert Swimmers; therefore, negative mean differences indicate higher accuracy in Expert Swimmers. Between-group comparisons were significant at all four difficulty levels: at Δ*t* = 200 ms, Mean Difference (MD) = −0.080, 95% *CI* [−0.137, −0.023], *t*(38) = −2.820, *p* = 0.030, Cohen's *d* = −0.892; at Δ*t* = 500 ms, *MD* = −0.196, 95% *CI* [−0.255, −0.136], *t*(38) = −6.671, *p* < 0.001, Cohen's *d* = −2.109; at Δ*t* = 1,000 ms, *MD* = −0.200, 95% *CI* [−0.267, −0.134], Welch's *t*(26.328) = −6.178, *p* < 0.001, Cohen's *d* = −1.954; and at Δ*t* = 2,000 ms, *MD* = −0.116, 95% *CI* [−0.163, −0.069], Welch's *t*(22.686) = −5.123, *p* < 0.001, Cohen's *d* = −1.620 (see Figure 2A).

Within-group Bonferroni-adjusted comparisons further clarified this interaction pattern. In Expert Swimmers, accuracy at Δ*t* = 200 ms was lower than at Δ*t* = 500 ms, *MD* = −0.194, 95% *CI* [−0.270, −0.118], *t*(19) = −5.342, *p* < 0.001, Cohen's *d* = −1.195; at Δ*t* = 1,000 ms, *MD* = −0.315, 95% *CI* [−0.355, −0.275], *t*(19) = −16.628, *p* < 0.001, Cohen's *d* = −3.718; and at Δ*t* = 2,000 ms, *MD* = −0.356, 95% *CI* [−0.396, −0.316], *t*(19) = −18.664, *p* < 0.001, Cohen's *d* = −4.173. Accuracy at Δ*t* = 500 ms was also lower than at Δ*t* = 1,000 ms, *MD* = −0.121, 95% *CI* [−0.178, −0.065], *t*(19) = −4.496, *p* = 0.001, Cohen's *d* = −1.005, and lower than at Δ*t* = 2,000 ms, *MD* = −0.162, 95% *CI* [−0.217, −0.108], *t*(19) = −6.237, *p* < 0.001, Cohen's *d* = −1.395. Accuracy at Δ*t* = 1,000 ms remained lower than at Δ*t* = 2000 ms, *MD* = −0.041, 95% *CI* [−0.068, −0.015], *t*(19) = −3.283, *p* = 0.023, Cohen's *d* = −0.734. In Amateur Controls, the comparison between Δ*t* = 200 ms and 500 ms was not significant, *MD* = −0.078, 95% *CI* [−0.136, −0.020], *t*(19) = −2.806, *p* = 0.068, Cohen's *d* = −0.627. All remaining pairwise comparisons were significant: Δ*t* = 200 ms vs. 1,000 ms, *MD* = −0.195, 95% *CI* [−0.267, −0.122], *t*(19) = −5.631, *p* < 0.001, Cohen's *d* = −1.259; Δ*t* = 200 ms vs. 2,000 ms, *MD* = −0.320, 95% *CI* [−0.388, −0.252], *t*(19) = −9.845, *p* < 0.001, Cohen's *d* = −2.201; Δ*t* = 500 ms vs. 1,000 ms, *MD* = −0.117, 95% *CI* [−0.182, −0.051], *t*(19) = −3.735, *p* = 0.008, Cohen's *d* = −0.835; Δ*t* = 500 ms vs. 2,000 ms, *MD* = −0.242, 95% *CI* [−0.298, −0.186], *t*(19) = −9.007, *p* < 0.001, Cohen's *d* = −2.014; and Δ*t* = 1,000 ms vs. 2,000 ms, *MD* = −0.125, 95% *CI* [−0.187, −0.063], *t*(19) = −4.239, *p* = 0.003, Cohen's *d* = −0.948.

#### 2.2.2 Reaction time

Reaction time was originally recorded by the program in seconds (s), and converted to milliseconds (ms) prior to the final statistical analyses for clarity of presentation. Descriptive statistics showed that reaction time decreased as task difficulty decreased in both groups. In Expert Swimmers, mean reaction time was 1,131 ms (*SD* = 388) at Δ*t* = 200 ms, 1,006 ms (*SD* = 304) at Δ*t* = 500 ms, 866 ms (*SD* = 253) at Δ*t* = 1,000 ms, and 737 ms (*SD* = 224) at Δ*t* = 2,000 ms. In Amateur Controls, the corresponding values were 817 ms (*SD* = 306), 841 ms (*SD* = 303), 764 ms (*SD* = 258), and 684 ms (*SD* = 235), respectively (see Table 2).

With Greenhouse–Geisser correction, the 2 × 4 mixed-design repeated-measures ANOVA revealed a significant main effect of Task Difficulty, *F*_(2.434, 92.499)_ = 41.475, *p* < 0.001, η*p*^2^ = 0.522. The main effect of Group was not significant, *F*_(1, 38)_ = 3.434, *p* = 0.072, η*p*^2^ = 0.083. However, the Group × Task Difficulty interaction was significant, *F*_(2.434, 92.499)_ = 9.626, *p* < 0.001, η*p*^2^ = 0.202.

In the statistical file, the between-group reaction-time contrasts were also reported in the direction of Amateur Controls minus Expert Swimmers; therefore, negative mean differences indicate longer reaction times in Expert Swimmers. Bonferroni-adjusted simple-effects analyses showed that a significant between-group difference emerged only at Δ*t* = 200 ms, *MD* = −314 ms, 95% *CI* [−537, −90], *t*(38) = −2.838, *p* = 0.029, Cohen's *d* = −0.898. No significant between-group differences were observed at Δ*t* = 500 ms, *MD* = −165 ms, 95% *CI* [−360, 29], *t*(38) = −1.724, *p* = 0.372, Cohen's *d* = −0.545; at Δ*t* = 1,000 ms, *MD* = −102 ms, 95% *CI* [−265, 62], *t*(38) = −1.257, *p* = 0.866, Cohen's *d* = −0.397; or at Δ*t* = 2,000 ms, *MD* = −53 ms, 95% *CI* [−200, 94], *t*(38) = −0.729, *p* = 1.000, Cohen's *d* = −0.231 (see Figure 2B).

Within-group Bonferroni-adjusted comparisons showed that, in Expert Swimmers, the difference between Δ*t* = 200 ms and 500 ms was not significant, *MD* = 125 ms, 95% *CI* [26, 223], *t*(19) = 2.646, *p* = 0.096, Cohen's *d* = 0.592. All other pairwise comparisons were significant: Δ*t* = 200 ms vs. 1,000 ms, MD = 265 ms, 95% *CI* [156, 374], *t*(19) = 5.088, *p* < 0.001, Cohen's *d* = 1.138; Δ*t* = 200 ms vs. 2,000 ms, *MD* = 394 ms, 95% *CI* [287, 502], *t*(19) = 7.684, *p* < 0.001, Cohen's *d* = 1.718; Δ*t* = 500 ms vs. 1,000 ms, *MD* = 140 ms, 95% *CI* [77, 204], *t*(19) = 4.638, *p* = 0.001, Cohen's *d* = 1.037; Δ*t* = 500 ms vs. 2,000 ms, *MD* = 270 ms, 95% *CI* [211, 328], *t*(19) = 9.658, *p* < 0.001, Cohen's *d* = 2.160; and Δ*t* = 1,000 ms vs. 2,000 ms, *MD* = 129 ms, 95% *CI* [67, 192], *t*(19) = 4.345, *p* = 0.002, Cohen's *d* = 0.971. In Amateur Controls, the comparisons between Δ*t* = 200 ms and 500 ms, *MD* = −24 ms, 95% *CI* [−88, 41], *t*(19) = −0.765, *p* = 1.000, Cohen's *d* = −0.171, between Δ*t* = 200 ms and 1,000 ms, *MD* = 53 ms, 95% *CI* [−5, 110], *t*(19) = 1.921, *p* = 0.419, Cohen's *d* = 0.430, and between Δ*t* = 500 ms and 1,000 ms, *MD* = 76 ms, 95% *CI* [10, 143], *t*(19) = 2.392, *p* = 0.163, Cohen's d = 0.535, were not significant. By contrast, the comparisons between Δ*t* = 200 ms and 2,000 ms, *MD* = 134 ms, 95% *CI* [61, 207], *t*(19) = 3.845, *p* = 0.007, Cohen's *d* = 0.860, between Δ*t* = 500 ms and 2,000 ms, *MD* = 157 ms, 95% *CI* [81, 234], *t*(19) = 4.291, *p* = 0.002, Cohen's *d* = 0.960, and between Δ*t* = 1,000 ms and 2,000 ms, *MD* = 81 ms, 95% *CI* [31, 131], *t*(19) = 3.393, *p* = 0.018, Cohen's *d* = 0.759, were significant.

### 2.3 Brief discussion

Experiment 1 examined suprasecond visual duration discrimination in a two-alternative forced-choice task under silent conditions. Accuracy varied reliably with Task Difficulty and showed a main effect of Group as well as a Task Difficulty by Group interaction. Across the four difficulty levels, defined by Δ*t* = 200 ms, 500 ms, 1,000 ms, and 2,000 ms, Expert Swimmers were more accurate than Amateur Controls in every condition. Within the Expert Swimmers, accuracy at Δ*t* = 200 ms and 500 ms was lower than in the easier conditions, and performance approached a plateau between Δ*t* = 1,000 ms and 2,000 ms. In contrast, Amateur Controls showed a stable graded pattern across all four difficulty levels. For reaction time, the main effect of Group was not significant, whereas the main effect of Task Difficulty and the Task Difficulty by Group interaction were significant. The only reliable group difference occurred at Δ*t* = 200 ms, where Expert Swimmers responded more slowly than Amateur Controls. Overall, both groups showed longer reaction time as Task Difficulty increased.

Because the interval range in the present study spans 2,000–7,000 ms, spontaneous chronometric counting should be considered as a plausible contributor to performance. Counting can increase accuracy and reduce variability, and it may also violate the scalar property of timing. Moreover, when methods to discourage counting are compared, an interference task tends to distort timing judgments and increase variability more than either a no counting instruction or articulatory suppression (Rattat and Droit-Volet, 2012). To reduce contamination of construct validity by counting, Riemer and colleagues modified the classical two interval discrimination paradigm by removing the pause between intervals and changing the judgment to whether a brief event occurred in the first or the second half of a reference duration. This modification was accompanied by counting related increases in reaction time and reduced the tendency for spontaneous counting when no counting related instructions were provided (Riemer et al., 2022). However, because Experiment 2 needed to remain comparable to Experiment 1 in visual materials and response rules, we did not substantively alter the two-alternative forced-choice structure. Given the suprasecond interval range used in Experiment 1, spontaneous chronometric counting remained a plausible alternative strategy. In Experiment 2, task-irrelevant rhythmic auditory stimulation was introduced as a background manipulation that may have increased attentional load and reduced reliance on internally generated counting, although the present design did not include a direct manipulation check of counting.

## 3 Experiment 2

### 3.1 Materials and methods

#### 3.1.1 Participants

The recruitment procedures, group assignment criteria, background data collection, screening procedures, and ethical protocols for Experiment 2 were identical to those used in Experiment 1. Experiment 2 involved a newly recruited sample and ultimately included 40 male participants, comprising 20 Expert Swimmers and 20 Amateur Controls. The mean age of the total sample was 19.82 ± 1.24 years. The expert swimmer group had a mean age of 20.08 ± 1.43 years, with an average of 11.42 ± 1.57 years of systematic swim training, a mean weekly sport-specific training volume of 11.20 ± 2.36 h, and a mean primary-event World Aquatics Points score of 699.95 ± 27.33. The amateur control group had a mean age of 19.59 ± 0.96 years, with an average of 1.88 ± 0.86 years of swimming experience and a mean weekly physical activity volume of 2.14 ± 1.44 h. Detailed participant characteristics are presented in Table 3.

**Table 3:** Participant characteristics for Experiment 2.

Group	Age	Gender	Expertise		
			**Experience (years)**	**Exercise (hours/week)**	**WA points**
Experts	20.08 ± 1.43	20M	11.42 ± 1.57	11.20 ± 2.36	699.95 ± 27.33
Amateurs	19.59 ± 0.96	20M	1.88 ± 0.86	2.14 ± 1.44	–
Total	19.82 ± 1.24	40M	–	–	–

Definitions of variables and abbreviations are identical to Table 1.

#### 3.1.2 Apparatus and stimuli

The programming framework, display apparatus, laboratory environment, keyboard-based response collection, visual stimulus parameters, and spatial layout in Experiment 2 were identical to those used in Experiment 1. The principal methodological difference between the two experiments was that Experiment 2 introduced an additional rhythmic auditory stimulus during the sequential presentation phase, thereby creating a suprasecond duration-discrimination task under concurrent visual–auditory stimulation.

The auditory stimuli were presented via PsychPortAudio in high-priority mode with stereo output at a sampling rate of 44.1 kHz. Each auditory event was a 1,000-Hz pure tone with a duration of 50 ms. The digital amplitude parameter was fixed at 0.3 for all trials. Four predefined rhythmic patterns were used. On each trial, one rhythm pattern was assigned to the blue interval and a different pattern was assigned to the yellow interval. Audio onset was triggered at the onset of each visual interval, and auditory onset timestamps were logged for trial-level verification of audiovisual timing. Auditory stimuli were delivered through BOSE QuietComfort 45 headphones.

#### 3.1.3 Procedure

Experiment 2 followed the same overall procedure as Experiment 1, including the standardized no-counting instruction, the 10-trial practice phase with feedback, the practice screening criteria, the response mapping (*F* = blue longer; *J* = yellow longer), the 4,000-ms response deadline, the 500-ms intertrial interval, and the post-task strategy check. The formal test again comprised 80 trials, with difficulty manipulated by four Δ*t* levels (200, 500, 1,000, and 2,000 ms; 20 trials per level) presented in randomized order.

**Figure 3:**
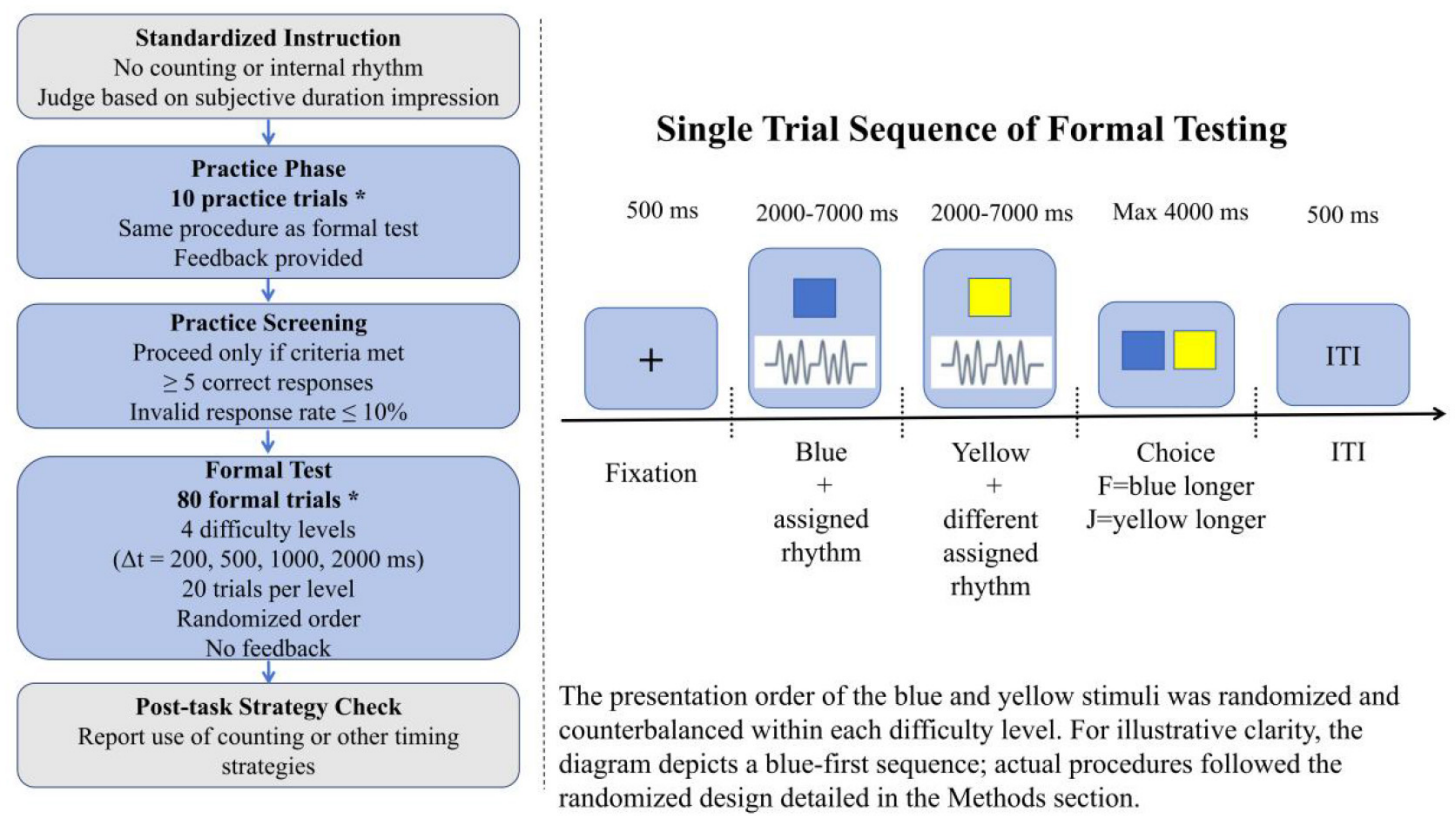
Procedure of Experiment 2 in the visual duration-discrimination task with concurrent rhythmic auditory stimulation. Experiment 2 retained the instruction, practice, screening, formal test, and post-task strategy-check structure of Experiment 1. The critical manipulation was that each sequentially presented visual interval was accompanied by rhythmic auditory stimulation, and in each trial the durations of the blue square and yellow square ranged from 2,000 to 7,000 ms. All other aspects were identical to Experiment 1.

The critical manipulation was the addition of rhythmic auditory stimulation during the sequential presentation phase. In each formal trial, a 500-ms fixation cross was followed by the sequential presentation of the two colored squares (with their presentation order randomized and counterbalanced, identical to Experiment 1), each with an individually generated duration constrained to 2,000–7,000 ms under the designated Δ*t* condition. During each visual interval, a rhythmic auditory sequence was played concurrently. The task program implemented four rhythm categories and, on each trial, assigned one rhythm ID to the blue interval and a different rhythm ID to the yellow interval, such that the two sequential intervals were always paired with different rhythms within the same trial. Participants then judged which interval had lasted longer. The auditory rhythm was embedded as an accompanying stimulus rather than as an independent task. In addition to the variables recorded in Experiment 1, the program logged the rhythm IDs assigned to the blue and yellow intervals and the corresponding auditory onset markers for each trial, thereby allowing trial-level verification of rhythm assignment and audiovisual timing. Pressing Escape during the choice phase terminated the task and saved all completed trial data automatically (see Figure 3).

#### 3.1.4 Data analysis

The data-cleaning procedure, descriptive statistics, distributional diagnostics, mixed-design repeated-measures ANOVA framework, sphericity testing and corresponding corrections, effect-size reporting conventions, and simple-effects analyses conducted when interactions were significant in Experiment 2 were identical to those used in Experiment 1. Accordingly, the purpose of the statistical analysis in the present experiment was not to alter the analytical framework itself, but rather to examine whether the pattern of effects associated with Group and Task Difficulty changed after the addition of rhythmic auditory stimulation.

### 3.2 Results

For Experiment 2, 48 individuals were initially screened. Of these, 1 was excluded for failing to meet the study's prespecified inclusion criteria, 4 did not meet the practice phase qualification criteria (2 excluded for substandard overall accuracy in the practice phase, 2 for failure to meet the prespecified reaction time criteria), 1 was excluded because overall accuracy in the formal task did not exceed chance level, 1 was excluded for failure to meet the prespecified reaction time criteria in the formal task, and 1 was excluded due to self-reported sustained active counting during the formal test. The final analyzed sample therefore comprised 20 expert swimmers and 20 amateur controls.

All analyses were based on 40 participants, including 20 Expert Swimmers and 20 Amateur Controls. Preliminary assumption checks indicated that the data were generally suitable for parametric analyses, although one accuracy cell showed substantial deviation from normality. Across all Group × Task Difficulty cells, skewness ranged from −2.068 to 0.802 and kurtosis ranged from −1.077 to 3.285 for accuracy, whereas skewness ranged from 0.288 to 1.455 and kurtosis ranged from −1.553 to 1.914 for reaction time. Mauchly's test indicated that the sphericity assumption was met for accuracy, *W* = 0.799, *p* = 0.144, but violated for reaction time, *W* = 0.398, *p* < 0.001; therefore, Greenhouse–Geisser correction was applied to the reaction time analyses. Levene's tests indicated homogeneous variances for accuracy at Δ*t* = 200 ms, 500 ms, and 1,000 ms, but unequal variances at Δ*t* = 2000 ms. For reaction time, the homogeneity assumption was met at all four difficulty levels.

#### 3.2.1 Accuracy

Descriptive statistics showed that Expert Swimmers were more accurate than Amateur Controls across all four difficulty levels. In Expert Swimmers, mean accuracy was 0.665 (*SD* = 0.088) at Δ*t* = 200 ms, 0.795 (*SD* = 0.072) at Δ*t* = 500 ms, 0.913 (*SD* = 0.086) at Δ*t* = 1,000 ms, and 0.988 (*SD* = 0.028) at Δ*t* = 2,000 ms. In Amateur Controls, the corresponding values were 0.595 (*SD* = 0.086), 0.668 (*SD* = 0.113), 0.783 (*SD* = 0.099), and 0.925 (*SD* = 0.066), respectively.

**Figure 4:**
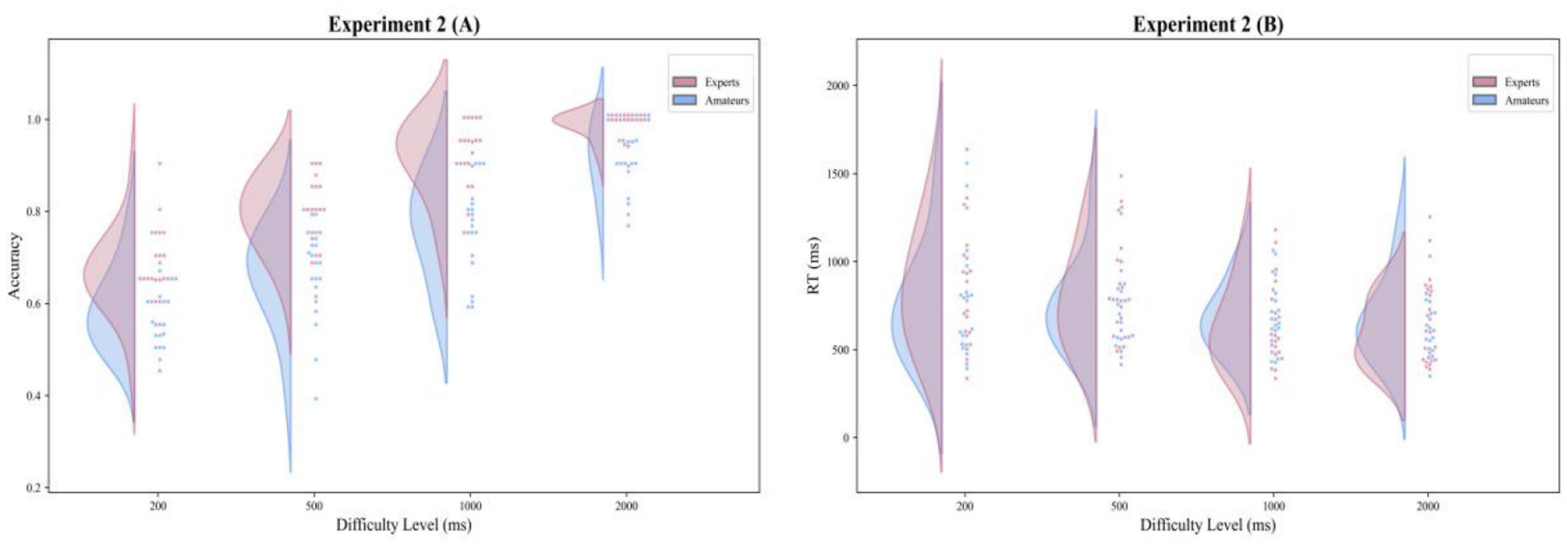
Experiment 2 (audiovisual) performance for experts and amateurs (*n* = 20 per group) across Δ*t* (200, 500, 1,000, 2,000 ms). **(A)** Accuracy. **(B)** Reaction time (ms; from choice-display onset); no significance markers are shown because follow-up simple effects were not conducted.

A 2 × 4 mixed-design repeated-measures ANOVA revealed a significant main effect of Group, *F*_(1, 38)_ = 50.042, *p* < 0.001, η*p*^2^ = 0.568, and a significant main effect of Task Difficulty, *F*_(2.651, 100.739)_ = 120.021, *p* < 0.001, η*p*^2^ = 0.760. The Group × Task Difficulty interaction was not significant, *F*_(2.651, 100.739)_ = 1.969, *p* = 0.131, η*p*^2^ = 0.049. Accordingly, no further simple-effects analyses were conducted for accuracy (see Table 4 and Figure 4A).

**Table 4:** Experiment 2 descriptive statistics for accuracy and reaction time.

Group	Δ*t* = 200 ms	Δ*t* = 500 ms	Δ*t* = 1,000 ms	Δ*t* = 2,000 ms
Accuracy (*n* = 20)				
Experts (*M* ±*SD*)	0.665 ± 0.088	0.795 ± 0.072	0.913 ± 0.086	0.988 ± 0.028
Amateurs (*M* ±*SD*)	0.595 ± 0.086	0.668 ± 0.113	0.783 ± 0.099	0.925 ± 0.066
Reaction time (*n* = 20)				
Experts (*M* ±*SD*)	863 ± 347	799 ± 285	638 ± 240	602 ± 186
Amateurs (*M* ±*SD*)	750 ± 315	747 ± 255	682 ± 190	670 ± 232

For the expert swimmer group and the amateur control group (*n* = 20 per group), data at each task difficulty level, indexed by Δ*t* (200, 500, 1,000, and 2,000 ms), are presented as mean ± standard deviation (*M* ± *SD*). All values are reported to three decimal places, and reaction times (RT) are reported in milliseconds. Under the audiovisual condition, each visual interval was accompanied by a task-irrelevant rhythmic auditory stimulus.

#### 3.2.2 Reaction time

Reaction time was originally recorded by the program in seconds (s), and converted to milliseconds (ms) prior to the final statistical analyses for clarity of presentation. Descriptive statistics showed that reaction time decreased as task difficulty decreased in both groups. In Expert Swimmers, mean reaction time was 863 ms (*SD* = 347) at Δ*t* = 200 ms, 799 ms (*SD* = 285) at Δ*t* = 500 ms, 638 ms (*SD* = 240) at Δ*t* = 1,000 ms, and 602 ms (*SD* = 186) at Δ*t* = 2,000 ms. In Amateur Controls, the corresponding values were 750 ms (*SD* = 315), 747 ms (*SD* = 255), 682 ms (*SD* = 190), and 670 ms (*SD* = 232), respectively (see Table 4).

With Greenhouse–Geisser correction, the 2 × 4 mixed-design repeated-measures ANOVA revealed no significant main effect of Group, *F*_(1, 38)_ = 0.030, *p* = 0.863, η*p*^2^ = 0.001. The main effect of Task Difficulty was significant, *F*_(1.838, 69.857)_ = 19.647, *p* < 0.001, η*p*^2^ = 0.341. Importantly, the Group × Task Difficulty interaction was also significant, *F*_(1.838, 69.857)_ = 5.021, *p* = 0.011, η*p*^2^ = 0.117.

Because the interaction was significant, Bonferroni-adjusted simple-effects analyses were conducted. In the statistical file, the between-group contrasts were reported in the direction of Amateur Controls minus Expert Swimmers; therefore, negative mean differences indicate longer reaction times in Expert Swimmers. However, no between-group comparison reached significance at any difficulty level: at Δ*t* = 200 ms, *MD* = −113 ms, 95% *CI* [−325, 99], *t*(38) = −1.077, *p* = 1.000, Cohen's *d* = −0.341; at Δ*t* = 500 ms, *MD* = −52 ms, 95% *CI* [−225, 121], *t*(38) = −0.610, *p* = 1.000, Cohen's *d* = −0.193; at Δ*t* = 1,000 ms, *MD* = 44 ms, 95% *CI* [−95, 182], *t*(38) = 0.639, *p* = 1.000, Cohen's *d* = 0.202; and at Δ*t* = 2,000 ms, *MD* = 68 ms, 95% *CI* [−66, 203], *t*(38) = 1.029, *p* = 1.000, Cohen's *d* = 0.325 (see Figure 4B).

Within-group Bonferroni-adjusted comparisons showed distinct patterns across groups. In Expert Swimmers, the difference between Δ*t* = 200 ms and 500 ms was not significant, *MD* = 64 ms, 95% *CI* [0, 128] ms, *t*(19) = 2.099, *p* = 0.297, Cohen's *d* = 0.469, and the difference between Δ*t* = 1,000 ms and 2,000 ms was also not significant, *MD* = 36 ms, 95% *CI* [−24, 96] ms, *t*(19) = 1.265, *p* = 1.000, Cohen's *d* = 0.283. By contrast, reaction time at Δ*t* = 200 ms was significantly longer than at Δ*t* = 1,000 ms, *MD* = 225 ms, 95% *CI* [123, 327] ms, *t*(19) = 4.607, *p* = 0.001, Cohen's *d* = 1.030, and at Δ*t* = 2,000 ms, *MD* = 261 ms, 95% *CI* [133, 388] ms, *t*(19) = 4.278, *p* = 0.002, Cohen's *d* = 0.957. Reaction time at Δ*t* = 500 ms was also significantly longer than at Δ*t* = 1,000 ms, *MD* = 161 ms, 95% *CI* [100, 222] ms, *t*(19) = 5.495, *p* < 0.001, Cohen's *d* = 1.229, and at Δ*t* = 2,000 ms, *MD* = 197 ms, 95% *CI* [111, 283] ms, *t*(19) = 4.791, *p* = 0.001, Cohen's *d* = 1.071. In Amateur Controls, none of the Bonferroni-adjusted within-group comparisons reached significance. Specifically, the comparisons between Δ*t* = 200 ms and 500 ms, *MD* = 3 ms, 95% *CI* [−62, 68] ms, *t*(19) = 0.103, *p* = 1.000, Cohen's *d* = 0.023; between Δ*t* = 200 ms and 1,000 ms, *MD* = 68 ms, 95% *CI* [−16, 152] ms, *t*(19) = 1.700, *p* = 0.632, Cohen's *d* = 0.380; between Δ*t* = 200 ms and 2,000 ms, *MD* = 80 ms, 95% *CI* [−6, 165] ms, *t*(19) = 1.957, *p* = 0.391, Cohen's *d* = 0.438; between Δ*t* = 500 ms and 1,000 ms, *MD* = 65 ms, 95% *CI* [2, 128] ms, *t*(19) = 2.162, *p* = 0.261, Cohen's *d* = 0.484; between Δ*t* = 500 ms and 2,000 ms, *MD* = 76 ms, 95% *CI* [21, 132] ms, *t*(19) = 2.862, *p* = 0.060, Cohen's *d* = 0.640; and between Δ*t* = 1,000 ms and 2,000 ms, *MD* = 12 ms, 95% *CI* [−47, 70] ms, *t*(19) = 0.414, *p* = 1.000, Cohen's *d* = 0.093, were all non-significant after Bonferroni correction.

## 4 Discussion

### 4.1 Principal findings

Across two independent experiments, the present study showed a consistent accuracy advantage for Expert Swimmers over Amateur Controls in a neutral visual suprasecond duration-discrimination task. In both experiments, discrimination performance improved as task difficulty decreased, indicating that the basic difficulty manipulation functioned as expected. Evidence for a greater expert advantage under higher difficulty was observed in Experiment 1 but was not reproduced in Experiment 2, suggesting that this aspect of the pattern was not fully stable across samples. Reaction time also decreased as task difficulty decreased; however, no overall group-related reaction-time advantage was observed. At the same time, the expertise-related accuracy advantage remained observable in both the silent visual context and the task-irrelevant rhythmic context, whereas group-related variation in reaction-time patterning was present but does not by itself warrant a strong mechanistic conclusion. Taken together, these findings indicate that, within the present task structure, swimming expertise was associated primarily with more accurate suprasecond duration discrimination rather than with a generalized speed benefit.

### 4.2 Relation to prior literature

The present findings are broadly consistent with prior evidence that extensive sport practice can be associated with superior temporal performance, but they also narrow the conditions under which such an advantage can be interpreted. Consistent with Chen and Cesari (2015), who reported more accurate and less variable temporal performance in elite athletes, and with Perrone et al. (2023), who found that closed-skill athletes—especially swimmers—showed more accurate and more consistent timing than non-athletes, Expert Swimmers in the present study showed higher accuracy than Amateur Controls. Importantly, however, the current study differs from those reports along the task axis: the present advantage emerged in a neutral visual suprasecond duration-discrimination task rather than in time reproduction, finger tapping, or motor imagery paradigms. This difference is theoretically consequential because swimmer advantages have often been demonstrated in tasks that preserved sport-related temporal structure or action content, such as task duration knowledge for familiar swimming actions (Tobin and Grondin, 2012) or temporal prediction in swimming-specific scenes (Bove et al., 2017). Relative to that literature, the present findings suggest that the swimmer advantage is not fully dependent on overt motor production, sport imagery, or swimming-specific semantics. This more conservative interpretation is also compatible with recent non-sport evidence showing that stable motor timing can selectively sharpen perceptual discrimination for matched temporal intervals (Guo et al., 2025).

A similar point emerges from comparison with Zheng (2024), who found athlete-related advantages for implied-motion stimuli primarily in the suprasecond range. Relative to that study, the present findings suggest that suprasecond athlete-related benefits can remain observable even when implied motion is removed and the stimuli are reduced to neutral visual intervals. At the same time, the present results should not be interpreted as evidence for a fully amodal timing advantage. Rather, they are better understood as evidence of bounded transfer within the present task structure, because modality-specific differences in temporal sensitivity remain robust in the broader literature, with auditory timing typically outperforming visual timing (Rammsayer et al., 2015; Cantarella et al., 2024). Finally, the persistence of the group difference in the task-irrelevant rhythmic context is consistent with evidence that rhythmic sound does not automatically facilitate visual task performance when it is neither attended nor predictive (De Winne et al., 2022). Taken together, these comparisons indicate that the present study extends the athlete-timing literature in a specific and theoretically important way: it identifies an expertise-related advantage in a neutral visual suprasecond comparison task and across two sensory contexts, while stopping short of claiming unrestricted cross-task or cross-modal generality.

### 4.3 Alternative explanations

The present findings support an expertise-related advantage within the current task structure, but they do not by themselves specify the level of processing at which that advantage arose. Because the task required explicit comparison of neutral visual intervals in the suprasecond range, the observed group difference may reflect not only temporal discrimination *per se*, but also processes that are closely involved in suprasecond judgment, including attentional allocation, working-memory maintenance, and interval comparison. This more cautious interpretation is consistent with classic time-perception accounts emphasizing the role of attention and memory in explicit duration judgments (Zakay and Block, 1997; Block et al., 2010; Grondin, 2010), and it is also compatible with more recent work suggesting that performance in the suprasecond range is especially susceptible to higher-order cognitive influences, including those associated with trained action contexts (Zheng, 2024; Teghil and Wittmann, 2025). Accordingly, the present group difference is better described as an expertise-related advantage in suprasecond temporal decision performance within the current visual comparison task, rather than as direct evidence for a selectively enhanced internal timing mechanism.

A similar caution is required for the reaction-time findings. In the present study, the RT pattern is compatible with the possibility that Expert Swimmers adjusted their response criterion more systematically as task difficulty increased, particularly under conditions of greater temporal uncertainty. However, RT and accuracy alone cannot determine whether the relevant group difference arose from stronger temporal evidence, more cautious responding, or another latent decision component. That distinction requires formal sequential-sampling approaches, such as diffusion-model analyses, rather than descriptive RT interpretation alone (Ratcliff and McKoon, 2008; Heitz, 2014). For this reason, the current RT results should be interpreted as behaviorally informative but mechanistically non-decisive.

Finally, the fact that the accuracy interaction was observed in Experiment 1 but not reproduced in Experiment 2 should not be taken as evidence that different timing mechanisms operated across the two experiments. A more conservative explanation is that, in this type of suprasecond visual discrimination task, accuracy is not equally sensitive to group differences across all difficulty regions. When performance approaches floor-adjacent or ceiling-adjacent levels, between-group differences may be compressed, whereas threshold-adjacent difficulty may reveal them more clearly. On this reading, the Experiment 1 interaction is more parsimoniously interpreted as a difference in measurement sensitivity across difficulty regions than as evidence of a qualitatively different underlying process. Taken together, these considerations suggest that the present results are most appropriately interpreted as evidence of an expertise-related advantage under the specific cognitive and perceptual demands of the current task, while leaving open multiple process-level explanations that require direct testing in future work.

### 4.4 Practical implications

From an applied perspective, the present findings support several cautious implications for coaching, sport-psychology support, and athlete development. For coaches and performance staff, performance on the present neutral visual suprasecond duration-discrimination task may be considered as a supplementary behavioral indicator of pacing-related temporal control and judgment stability under graded difficulty, particularly when interpreted longitudinally and relative to athlete-specific baselines. For sport-psychology support, the pattern of higher accuracy without a generalized reaction-time advantage suggests that this task may be more useful for contextualizing attentional stability and response consistency under temporal uncertainty than for evaluating response speed alone. In athlete development settings, the present results do not justify the use of this task as a stand-alone selection tool. Rather, if future prospective work establishes predictive validity, it may be more appropriately considered as one auxiliary profiling variable within a broader, multidimensional evaluation framework.

### 4.5 Limitations and future directions

Several limitations should be acknowledged. First, although Experiment 2 introduced rhythmic sound, the manipulation was task-irrelevant and non-predictive. As a result, the present study could not directly test whether rhythm would influence performance when it is attended, behaviorally relevant, or temporally informative. Future work should manipulate these components more explicitly, because perceptual benefits from rhythmic structure appear to depend not only on regularity itself but also on temporal expectation and predictability (De Winne et al., 2022; Heynckes et al., 2023). Second, the present interpretation of the reaction-time pattern remained descriptive. Because RT and accuracy were not analyzed with a formal sequential-sampling framework, the current data cannot distinguish whether the observed group-related differences were driven primarily by changes in evidence quality, response caution, or other latent decision components (Ratcliff and McKoon, 2008; Heitz, 2014). Third, the study relied exclusively on behavioral measures. Incorporating neural or physiological indices in future work would help clarify whether the observed advantage is more closely related to visual timing, temporal expectation, or broader attentional regulation, which cannot be fully dissociated on the basis of behavior alone (Nobre, 2023; Yu et al., 2022). A further limitation concerns the control of counting-related strategies. Although participants received standardized no-counting instructions and completed a post-task strategy check, the suprasecond interval range used here makes it difficult to exclude counting completely. Moreover, the rhythmic auditory background in Experiment 2 was not validated as a direct anti-counting manipulation. Accordingly, the present findings should be interpreted as reflecting expertise-related differences in task performance under the current strategy-control procedure, rather than as process-pure evidence of a counting-free timing mechanism. Finally, the present findings were derived from a cross-sectional group comparison and therefore do not address whether improving performance on this type of suprasecond temporal judgment task would translate into measurable gains in sport performance. Future intervention-based studies could usefully examine whether targeted training in temporal judgment stability under uncertainty is associated with changes in pacing-related control or other performance-relevant outcomes, an issue that remains important in the broader perceptual-cognitive intervention literature (Bernier et al., 2025; Zhu et al., 2024).

## 5 Conclusion

Across two independent experiments, the present study examined whether swimming expertise is associated with superior performance in a neutral visual suprasecond duration-discrimination task under silent and task-irrelevant rhythmic contexts. Expert Swimmers showed higher accuracy than Amateur Controls across difficulty levels in both experiments, whereas reaction-time findings did not indicate a generalized speed advantage. The presence of task-irrelevant rhythmic sound did not materially alter the overall accuracy pattern, indicating that the observed expertise-related advantage was not dependent on immediate facilitation from external rhythm. Taken together, these findings support the view that swimming expertise is associated with more accurate suprasecond visual duration discrimination within the present task structure, while also suggesting that this advantage extends beyond overtly sport-specific stimulus content. At the same time, the present evidence is better interpreted as support for bounded transfer within a neutral visual comparison context than as proof of unrestricted domain-general timing. Future work should combine more targeted manipulations of rhythmic relevance and predictability with formal decision modeling and intervention-based designs to clarify both the mechanisms and the practical significance of expertise-related temporal advantages.

## Data Availability

The raw data supporting the conclusions of this article will be made available by the authors, without undue reservation.
